# Genome Based Phylogeny and Comparative Genomic Analysis of Intra-Mammary Pathogenic *Escherichia coli*

**DOI:** 10.1371/journal.pone.0119799

**Published:** 2015-03-25

**Authors:** Vincent P. Richards, Tristan Lefébure, Paulina D. Pavinski Bitar, Belgin Dogan, Kenneth W. Simpson, Ynte H. Schukken, Michael J. Stanhope

**Affiliations:** 1 Department of Population Medicine and Diagnostic Sciences, College of Veterinary Medicine, Cornell University, Ithaca, New York, United States of America; 2 Department of Clinical Sciences, College of Veterinary Medicine, Cornell University, Ithaca, New York, United States of America; Wilfrid Laurier University, CANADA

## Abstract

*Escherichia coli* is an important cause of bovine mastitis and can cause both severe inflammation with a short-term transient infection, as well as less severe, but more chronic inflammation and infection persistence. *E*. *coli* is a highly diverse organism that has been classified into a number of different pathotypes or pathovars, and mammary pathogenic *E*. *coli* (MPEC) has been proposed as a new such pathotype. The purpose of this study was to use genome sequence data derived from both transient and persistent MPEC isolates (two isolates of each phenotype) to construct a genome-based phylogeny that places MPEC in its phylogenetic context with other *E*. *coli* pathovars. A subsidiary goal was to conduct comparative genomic analyses of these MPEC isolates with other *E*. *coli* pathovars to provide a preliminary perspective on loci that might be correlated with the MPEC phenotype. Both concatenated and consensus tree phylogenies did not support MPEC monophyly or the monophyly of either transient or persistent phenotypes. Three of the MPEC isolates (ECA-727, ECC-Z, and ECA-O157) originated from within the predominately commensal clade of *E*. *coli*, referred to as phylogroup A. The fourth MPEC isolate, of the persistent phenotype (ECC-1470), was sister group to an isolate of ETEC, falling within the *E*. *coli* B1 clade. This suggests that the MPEC phenotype has arisen on numerous independent occasions and that this has often, although not invariably, occurred from commensal ancestry. Examination of the genes present in the MPEC strains relative to the commensal strains identified a consistent presence of the type VI secretion system (T6SS) in the MPEC strains, with only occasional representation in commensal strains, suggesting that T6SS may be associated with MPEC pathogenesis and/or as an inter-bacterial competitive attribute and therefore could represent a useful target to explore for the development of MPEC specific inhibitors.

## Introduction

Dairy cattle mastitis, or inflammation of the mammary gland, is the dominant bovine health disorder affecting milk production worldwide, with reported annual losses exceeding two billion dollars in the United States dairy industry [1–3]. A variety of both gram positive and negative species of bacteria, including *Escherichia coli* [4], are linked to bovine mastitis. *E*. *coli* can cause both severe inflammation with a short-term transient infection, as well as less severe but more chronic inflammation and infection persistence [5, 6]. *E*. *coli* is a highly diverse organism that has been classified into a number of different pathotypes or pathovars, including adherent-invasive *E*. *coli* (AIEC), avian pathogenic *E*. *coli* (APEC), enteroaggregative *E*. *coli* (EAEC), enterohemorrageic *E*. *coli* (EHEC), enteropathogenic *E*. *coli* (EPEC), enterotoxigenic *E*. *coli* (ETEC), extra-intestinal pathogenic *E*. *coli* (ExPEC), and uropathogenic *E*. *coli* (UPEC). Mammary pathogenic *E*. *coli* (MPEC) has been proposed as a new *E*. *coli* pathotype [4]. Distinct subspecies or phylogroups of *E*. *coli* have long been acknowledged [7–10] and strains of the different phylogroups appear to differ in their ecological niches, and disease characteristics. At present, there is no published *E*. *coli* phylogeny that includes MPEC isolates in a comparative dataset that encompasses representative isolates of the various *E*. *coli* pathovars. The only published sequence based phylogeny regarding MPEC that we are aware of, uses MLST data and reports a single most parsimonious tree of a set of 165 MP trees and includes only one non-MPEC isolate in the analysis [11]. There are studies involving MPEC isolates that employ a PCR based test [12] to assess *E*. *coli* phylogroup membership, however, this is not a phylogenetic analysis, and provides no information on relative relationships of the isolates within each phylogroup. Furthermore, this PCR based test has been shown to provide unreliable designations for certain phylogroups [13], and in particular, that group (phylogroup A) to which MPEC isolates are most typically assigned.

We have developed a collection of *E*. *coli* isolates that can be divided into two distinct groups: those isolated from intramammary infections (IMI) displaying transient infection characteristics (see for examples [14, 15]) and those isolated from IMI associated with persistently infected mammary glands (see for examples [16]). The purpose of this study is to use genome sequence data derived from both transient and persistent MPEC isolates to construct a genome-based phylogeny that places MPEC in its phylogenetic context with other *E*. *coli* pathovars, and to evaluate whether there is possibly a different evolutionary history underlying transient and persistent phenotypes. A subsidiary goal is to conduct comparative genomic analyses of these MPEC isolates with other *E*. *coli* pathovars to provide a preliminary perspective on loci that might be correlated with the MPEC phenotype.

## Materials and Methods

### Genome sequencing and annotation of MPEC strains

Two *E*. *coli* strains associated with transient IMI, and two strains associated with persistent IMI were evaluated. The strains from transient infections were ECA-727 [17] and ECA-O157 [18]. The strains ECA-727 and ECA-O157 have been repeatedly shown to cause only brief transient IMI following an intramammary challenge. The *E*. *coli* isolates associated with persistent IMI, strain ECC-Z [19] and strain ECC-1470 [5], were isolated from persistently infected cows. Presence of a persistent IMI was confirmed by pulsed-field gel electrophoresis (PFGE) typing of isolates collected over time from the same infected mammary quarter. All four strains have also been used in phenotypic studies of infection characteristics in mammary epithelial cells [5, 6].

We determined the genome sequences of the four MPEC strains isolated from transient (ECA-O157 and ECA-727) and persistent (ECC-1470 and ECC-Z) bovine mastitis. Roche/454 pyrosequencing, involving single or paired-end reads from the FLX sequencer, was used to determine the sequence of the four MPEC genomes. The genome sequence for ECA-727 did not involve paired end data. The sequences were assembled (*De novo* assembly with Newbler Software) into 1173, 539, 994, and 24 contigs for ECA-0157, ECA-727, ECC-1470, and ECC-Z, respectively. Genome annotation for the four MPEC strains was done by the National Center for Biotechnology Information (NCBI) Prokaryotic Genomes Automatic Annotation Pipeline (PGAAP) at http://www.ncbi.nlm.nih.gov/genomes/static/Pipeline.html. The whole genome shotgun projects for ECA-0157, ECA-727, ECC-1470, and ECC-Z have been deposited at DDBJ/EMBL/GenBank under the accession numbers AHHK00000000, NZ_AHHL00000000, NZ_AHHM00000000, and AHHN00000000, respectively. We compared these four MPEC genome sequences to 56 complete *E*. *coli* genome sequences representing a combination of commensal isolates and the following ten pathotypes or pathovars: ABU (Asymptomatic Bacteriuria), AIEC, APEC, EAEC, EHEC, EPEC, ETEC, ExPEC, NMEC (Neonatal Meningitis), and UPEC. Also included were nine *Shigella* sp. isolates representing four different species. All of these 65 sequences were obtained from NCBI; their strain names, accession numbers, genome lengths and respective pathotypes are presented in S1 Table in the supporting information.

### Gene clustering and phylogenetic analyses

Homologous genes among the 69 genomes analyzed were delineated using the MCL algorithm [20] as implemented in the MCLBLASTLINE pipeline (available at http://micans.org/mcl). The pipeline uses Markov clustering (MCL) to assign genes to homologous clusters based on a BLASTp search between all pairs of protein sequences using an *E* value cut-off of 1e-5. The MCL algorithm was implemented using an inflation parameter of 1.8. Simulations have shown this value to be generally robust to false positives and negatives [21]. Nucleotide sequences corresponding to each MCL gene cluster were aligned using Probalign v1.1 [22]. For the phylogenetic analyses we selected those MCL gene clusters that were shared among all genomes and contained only single gene copies for each (the core set) (984 clusters). Recombination for genes in these clusters was assessed using a combination of three methods: (i) the Pairwise Homoplasy Index (PHI), (ii) the Neighbor Similarity Score (NSS), and (iii) Maximum χ^2^. We excluded any cluster that showed evidence of recombination for any two of the three methods. Using this approach, we excluded 546 clusters (438 remained).

We utilized two approaches to phylogenetic reconstruction. The first was a gene tree consensus approach. Here, phylogenetic trees for each MCL gene cluster alignment were obtained using maximum likelihood (PhyML v.3.0), and a consensus of these gene trees obtained using the Triple Construction Method as implemented in the program Triplec [23]. This procedure is based on the observation that the most probable three-taxon tree consistently matches the species tree [24]. The method searches all input trees for the most frequent of the three possible rooted triplets for each set of three taxa. Once found, the set of rooted triples are joined to form the consensus tree using the quartet puzzling heuristic [25]. The method has been shown to outperform majority-rule and greedy consensus methods [26]. The second approach was to generate an alignment of single nucleotide polymorphism (SNP) genotypes for each isolate by concatenating the aligned non-recombinant core gene clusters (16,062 SNPs). Phylogenetic analysis was again performed using maximum likelihood (PhyML v.3.0) [27]. Analyses were performed using the GTR+G substitution model, which was determined to be the best fit for the data using the Akaike Information Criterion (AIC) in MODELTEST [28]. Node support was obtained using non-parametric bootstrapping (500 replicates).

Gene Ontology (GO) terms were assigned to all genomes using Blast2GO v.2.5.0 [29]. Relative enrichment (over representation) of GO terms among lineages was assessed using Fisher exact tests. The test was performed using the Gossip statistical package [30] implemented within Blast2GO. The false discovery rate (FDR) procedure of Benjamini and Hochberg [31] was used to correct for multiple hypothesis testing (*FDR* = 0.05).

## Results and Discussion

Basic genome statistics for the four sequenced MPEC strains are summarized in Table 1.

*Escherichia coli* strains have been classified into an array of different phylogenetic groups, often referred to as phylogroups or phylotypes (A, B1, B2, C, D, E, F) [7–10], as well as a number of pathotypes (or pathovars), reflecting their different features of pathogenesis [32]. The Clermont genotyping scheme [12] has been used in several studies to assess the phylogroups of MPEC isolates and typically a majority, or at least a significant proportion, fall within phylogroup A, which includes predominately commensal isolates. For example, in a study from Iran about 45% of the isolates were classified as phylogroup A [33] and in another study from Finland about 83% were phylogroup A [34]. Recently, a study from China found 36% of clinical and subclinical bovine mastitis isolates were from group A, 58% group B1, and 6% group D [35]. Gordon et al. [13] compared triplex phylogroup determinations with MLST genotype data for a large number of isolates and determined that although overall the Clermont test correctly identified phylogroups 80–85% of the time, this differed depending on the particular phylogroup in question. Specifically, phylogroup A was seldom assigned correctly. This appears to be because the Clermont genotype of phylogroup A is the absence of all three PCR products, and because of potential mismatch between primer and template, absence of a PCR product in any diagnostic test is typically more unreliable than its presence. Gordon et al. suggested that strains failing to yield any PCR product are seldom members of phylogroup A and strains with such a genotype should not be assigned to a phylogroup. Therefore, Clermont determinations of MPEC as typically phylogroup A may not have been accurate.

**Table 1:** Basic genome statistics for the four MPEC strains.

Strain	Genome size (bp)	CDS	% GC	tRNAs
ECA-O157	4,654,138	5,179	50.7	47
ECA-727	4,859,863	4,901	50.9	50
ECC-1470	4,739,402	5,142	50.7	56
ECC-Z	4,911,586	4,679	53.0	69

**Fig 1:**
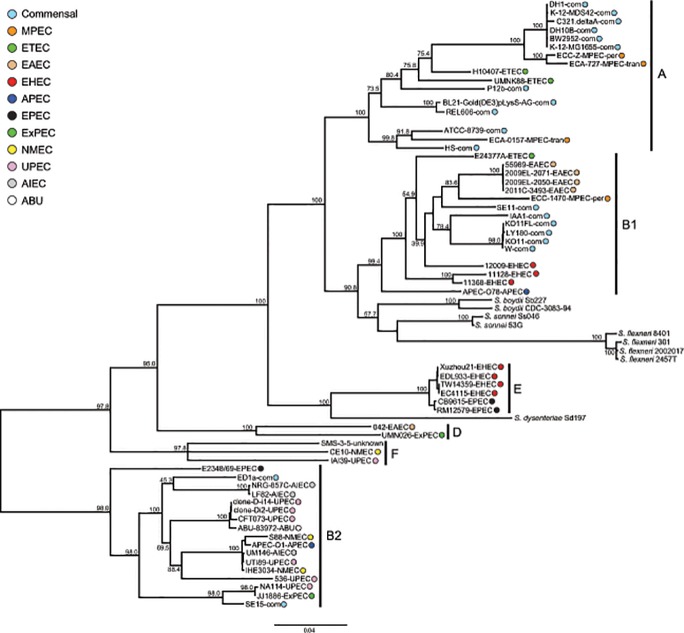
Maximum likelihood phylogeny generated from 16,062 SNPs. Bootstrap support values are shown over branches. Branch lengths are drawn proportion to the amount of sequence change. Phylogroup membership is indicated with a horizontal bar and the corresponding letter. Pathotypes of each of the *E*. *coli* strains is indicated with a color code and their lettered abbreviation adjacent to the strain names of each; commensal strains are abbreviated as “com”.

**Fig 2:**
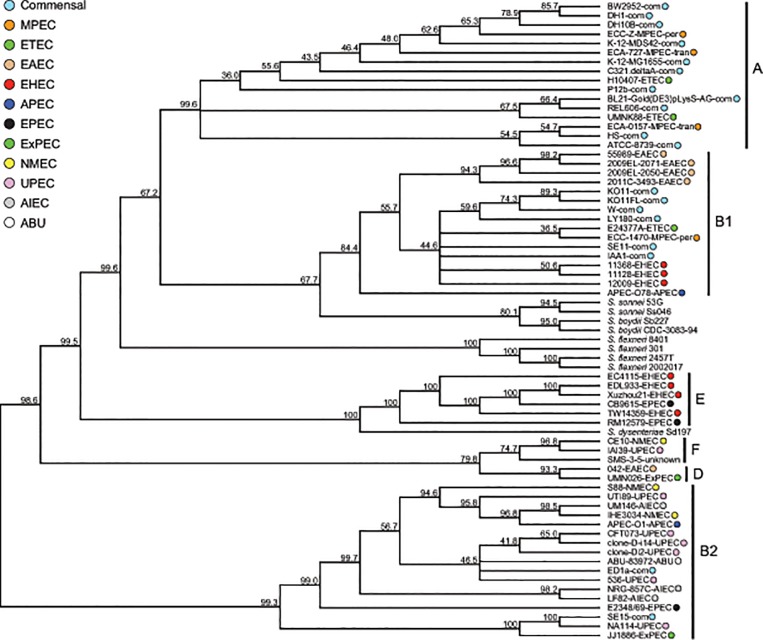
Consensus of 438 maximum likelihood gene phylogenies. Proportion of genes supporting groups shown on branches. Phylogroup and pathotype information same as in Fig. 1.

In the present study, we used a set of 438 orthologous loci to infer the evolutionary history of 60 *E*. *coli* strains, and nine strains of *Shigella*. This involved data for 56 complete genomes of *E*. *coli* taken from GenBank representing all the major phylotypes (with the exception of group C for which there were no complete genome sequences at the time this study was conducted) and pathovars, plus draft genome sequences of our four MPEC isolates. The comparative genome sequence data were first screened for putative recombinant loci, which were excluded in the phylogenetic reconstructions and therefore these orthologous loci should represent our best source of data to estimate the true evolutionary history of these set of isolates. We reconstructed the phylogeny using both a concatenated dataset that included all 438 of these non-recombinant orthologs (16,062 SNPs) (Fig. 1), and a consensus of the set of gene trees (Fig. 2). Both resulting trees were highly congruent and in agreement with earlier *E*. *coli* phylogenies (e.g. [36]), provided convincing support for the monophyly of the various phylogroups. Both the concatenated and the consensus trees did not support MPEC monophyly or the monophyly of either transient or persistent phenotypes. Three of these isolates (ECA-727, ECC-Z, and ECA-0157) originated from within the predominately commensal clade, referred to as phylogroup A. This classification is in accordance with our previous work, based on triplex PCR typing [5]. In the consensus phylogeny, ECC-Z, and ECA-727 were imbedded within a clade of six other exclusively commensal isolates. In the concatenated phylogeny these two MPEC isolates are a sister group to a clade of these same six commensal strains, which in turn are highly similar, as evidenced by the lack of branch length in Fig. 1. The branch length separating this monophyletic group of MPEC/commensal strains is the longest in phylogroup A, suggesting this MPEC/commensal group is quite distinct from the rest of the phylogroup. The phylogenetic position of ECA-O157 also suggests its origins are from commensal strains. The fourth MPEC isolate, of the persistent phenotype (ECC-1470), was sister group to an isolate of ETEC (E24377A) in the consensus phylogeny, falling within the B1 clade. This isolate was previously identified as belonging to phylogroup A [5], however, this classification was based on the absence of bands in a triplex PCR, which is more likely to be erroneous. In addition, none of the 438 individual ML trees that comprise the ultimate consensus tree supported the monophyly of MPEC. This then strongly suggests that the MPEC phenotype has arisen on at least three independent occasions and furthermore, that at least some of the time, this occurred from commensal ancestry. The fact that MPEC strains have a history that includes independent origin from commensal ancestry suggests that these isolates have acquired the loci underlying their pathogenic properties via lateral gene transfer (LGT). A comparison of the gene content of our two higher quality MPEC genome sequences (ECC-Z and ECA-727) relative to the 11 commensal strains that comprise the phylogroup A clade of our dataset, indicated that the MPEC strains are enriched, or over-represented, for the Genome Ontology (GO) categories of conjugation, and viral capsid assembly (FDR corrected P values of 3.26E-03 and 8.29E-04, respectively). They were under-represented for the categories of transposase activity, DNA mediated transposition, transposition, and DNA recombination (FDR corrected P values of 5.31E-19, 6.80E-18, 6.06E-13, 1.14E-06, respectively).

Earlier studies involving PCR surveys or DNA microarrays of *E*. *coli* virulence genes, and including many more MPEC isolates than are included here, have indicated that the presence and combinations of *E*. *coli* virulence loci in MPEC strains varies greatly (e.g. [11, 33, 34, 37]). Dogan et al. [37] showed that both transient and persistent MPEC isolates contained genes encoding type II, IV and VI secretion systems, long polar fimbriae (lpfA) and iron acquisition as potential pathogenicity loci. Since our isolate sample is limited, combined with the relatively low sequence coverage for two of our strains, we suggest there is little to be gained here by cataloging the presence and absence of virulence loci. Of somewhat greater possible interest, since our study does represent one of the first comparative genomic datasets involving MPEC strains, are the genes that are uniquely present in all four MPEC strains, or at least three of the four MPEC strains, and/or loci that are uniquely present in each of the transient or persistent strains (presented in S2 and S3 Table respectively). There was only one hypothetical protein unique to all four MPEC strains. Loci uniquely present in at least 3 MPEC strains included glutamate decarboxylase, multidrug efflux system protein MdtE, RhsC core protein with extension, a sensor protein, phosphoenolpyruvate synthase, an oxidoreductase, transcription-repair coupling factor, putative hydrolase, carboxymethylenebutenolidase and a few hypothetical proteins (S2 Table). The genes uniquely shared in the two transient MPEC strains included a set of 36 loci, whereas the genes uniquely present in the two persistent strains was only 11, and in this latter case, nearly all of which were hypothetical proteins (S3 Table). This transient and persistent comparison, however, should be regarded with a degree of skepticism because of the small sample size representing the two phenotypes and because of the relatively lower sequence coverage for two of the isolates (one persistent and one transient), which will in particular, make the assessment of absent loci somewhat tenuous.

Examination of the genes present in the MPEC strains relative to the commensal strains identified a consistent presence of the type VI secretion system (T6SS) in the MPEC strains, with more occasional representation in commensal strains (36.8% of strains). The type VI secretion system is the most recently described [38, 39] of the gram-negative bacterial secretion systems and is present in a diverse array of species. T6SSs inject effector proteins into both eukaryotic and prokaryotic target cells using a bacteriophage-like cell-puncturing device [40]. Targeting eukaryotic cells is part of the infection process and targeting prokaryotic cells is part of inter-bacterial antagonism and competition for the colonization of their environment. The effector proteins of these secretion systems are often critical for virulence; for example, the loss of the T3SS in *Yersinia pestis* is sufficient to render the bacteria completely avirulent [41].

**Fig 3:**
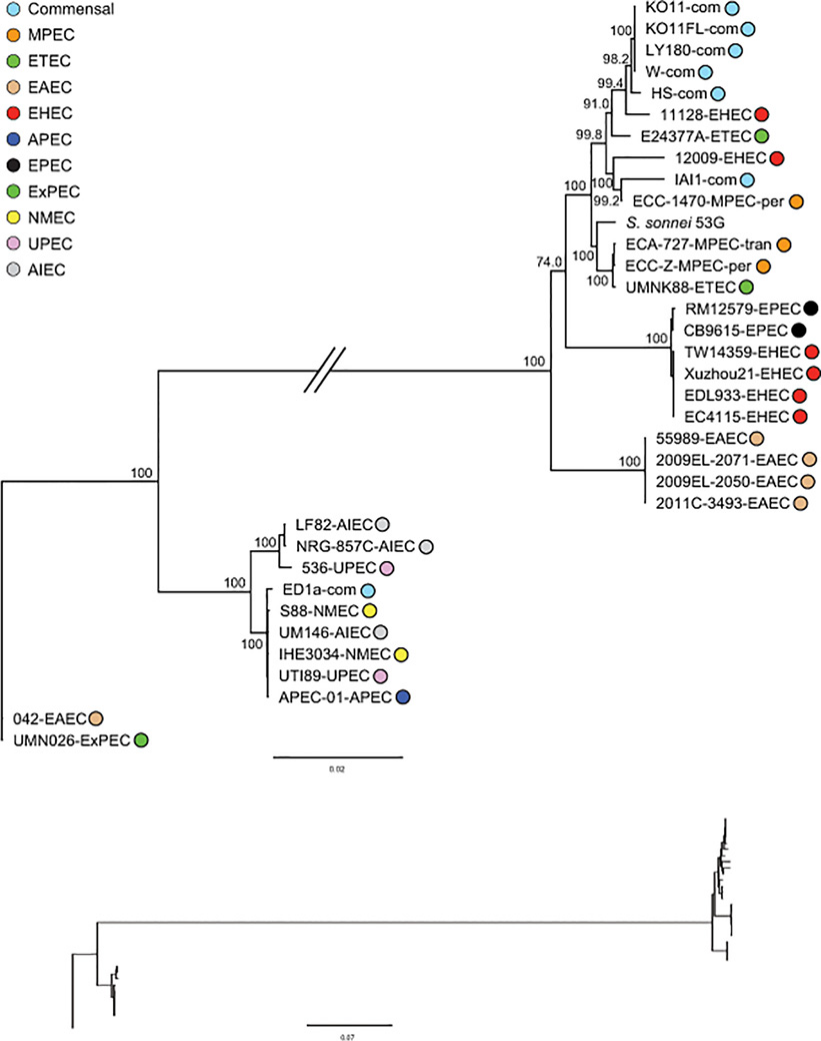
Maximum likelihood phylogeny for type six secretion system. Bootstrap support values are shown over branches. Branch lengths drawn proportion to the amount of sequence change. Phylogroup and pathotype information same as in Fig. 1.

The T6SS has been reported in commensal *E*. *coli*, including for example, strains W and HS included in our analyses [42]. It has also been identified in *E*. *coli* strains isolated from the ileum of patients with inflammatory bowel disease [43] including *E*. *coli* with an adherent-invasive (AIEC) pathotype. PCR based analysis of *E*. *coli* associated with mastitis, with similar adherent and invasive activity to AIEC, identified the T6SS *hcp* gene, a T6SS effector protein, in 88% of transient and 67% of persistent strains [37]. We conducted a BLASTn search including our MPEC isolates and the complete genome sequences included in our phylogeny, using the T6SS from strain W, which is referred to as the “enterohaemorrhagic” *E*. *coli* type six secretion system cluster [42, 44]. We found T6SS in all four of our MPEC strains, as well as 31 additional *E*. *coli* strains from the total of 69 strains included in Figs. 1 and 2. T6SS was present in only one of the commensal strains from phylogroup A and this was strain HS. For reference, S4 Table shows the locus_tags and gene product descriptions for the T6SS from strain W and the MPEC strain ECC-Z. For MPEC strain ECA-727, the T6SS spanned three contigs, with the majority of genes (15) present on one contig. We performed a global alignment of the genes from this contig and the corresponding orthologs from each of the 31 additional *E*. *coli* and the MPEC strains. MPEC strain ECA-O157 was excluded because of fragmentation across multiple different contigs. Using this alignment, we performed a ML phylogenetic analysis using PhyML (see methods). The GTR+G substitution model was determined as the best fit for the data and node support was obtained using non-parametric bootstrapping (500 replicates). The resulting phylogeny is presented in Fig. 3. The 5 additional commensal strains that had T6SS came from phylogroup B1. The fact that phylogroup A commensals in our data set do not tend to have T6SS, and that three of our four MPEC isolates originated from within phylogroup A, suggests that the MPEC isolates likely obtained T6SS via LGT. An examination of the loci up and downstream of the T6SS indicates the presence of various transposase and/or phage genes, in both our MPEC isolates as well as other isolates carrying T6SS. The T6SS phylogeny supports a clear dichotomy with two very different T6SS clades (Fig. 3) and these two groups tend to include isolates of distinct phylotypes and pathotypes. One of the T6SS groups includes representatives from predominately phylogroup B2 and the other from largely phylogroup B1, with a few phylogroup A representatives. Two of our MPEC strains share a close sister group relationship of their T6SS with a porcine enterotoxigenic *E*. *coli* (UMNK88-ETEC). The T6SS sequence similarity is very high between the ETEC and these two MPEC strains (ECA-727; ECC-Z). For example, after exclusion of a 1,619bp indel within the IcmF-like protein for strain ECC-Z, identity for the 15 genes used in the phylogeny was 99.9%. This high sequence identity occurred despite the fact these strains are not closely related in the strain phylogeny (Figs. 1 and 2), suggesting a possible LGT event involving the MPEC strains and this porcine ETEC or one closely related to it; these two MPEC strains include one persistent and one transient, with the T6SS for the other persistent strain (ECC-1470) not particularly closely related to either of these. Examination of the loci up and downstream of the T6SS in ECC-Z (isolate with the longest contig for that region), indicate a close synteny between the MPEC and ETEC strains, including transposase. This evolutionary picture then, suggests a T6SS LGT event involving an ETEC strain, possibly UMNK88, or a closely related strain to UMNK88. Furthermore, these data suggest that T6SS may well be associated with MPEC strains, either as a specific feature of pathogenesis and/or as an inter-bacterial competitive attribute, but possibly not specifically related to differences between transient and persistent phenotypes. Further research on the impact of T6SS on pathogenesis of bovine mastitis in challenge models comparing T6SS mutants and wild types appears indicated.

## Supporting Information

S1 Table Strain names, accession numbers, genome sequence length and pathotype for each isolate included in this study, as well as sequencing statistics for the four MPEC strains. (XLSX)

S2 Table Genes that are uniquely present in at least three of the four MPEC strains in comparisons to the other genomes in our dataset. (XLSX)

S3 Table Genes that are uniquely present in the transient and persistent MPEC strains in comparisons to the other genomes in our dataset. (XLSX)

S4 Table Locus tags, gene product descriptions, and gene sequence identities for the T6SS from strain W and the MPEC strain ECC-Z. (XLSX)
