# Correction: Genome Based Phylogeny and Comparative Genomic Analysis of Intra-Mammary Pathogenic *Escherichia coli*

**DOI:** 10.1371/journal.pone.0133222

**Published:** 2015-07-14

**Authors:** The PLOS ONE Staff

The image quality in Fig 1, Fig 2, and Fig 3 is poor. Please see below for the correct versions. The journal apologizes for these errors, which were introduced during the typesetting process.

**Fig 1:**
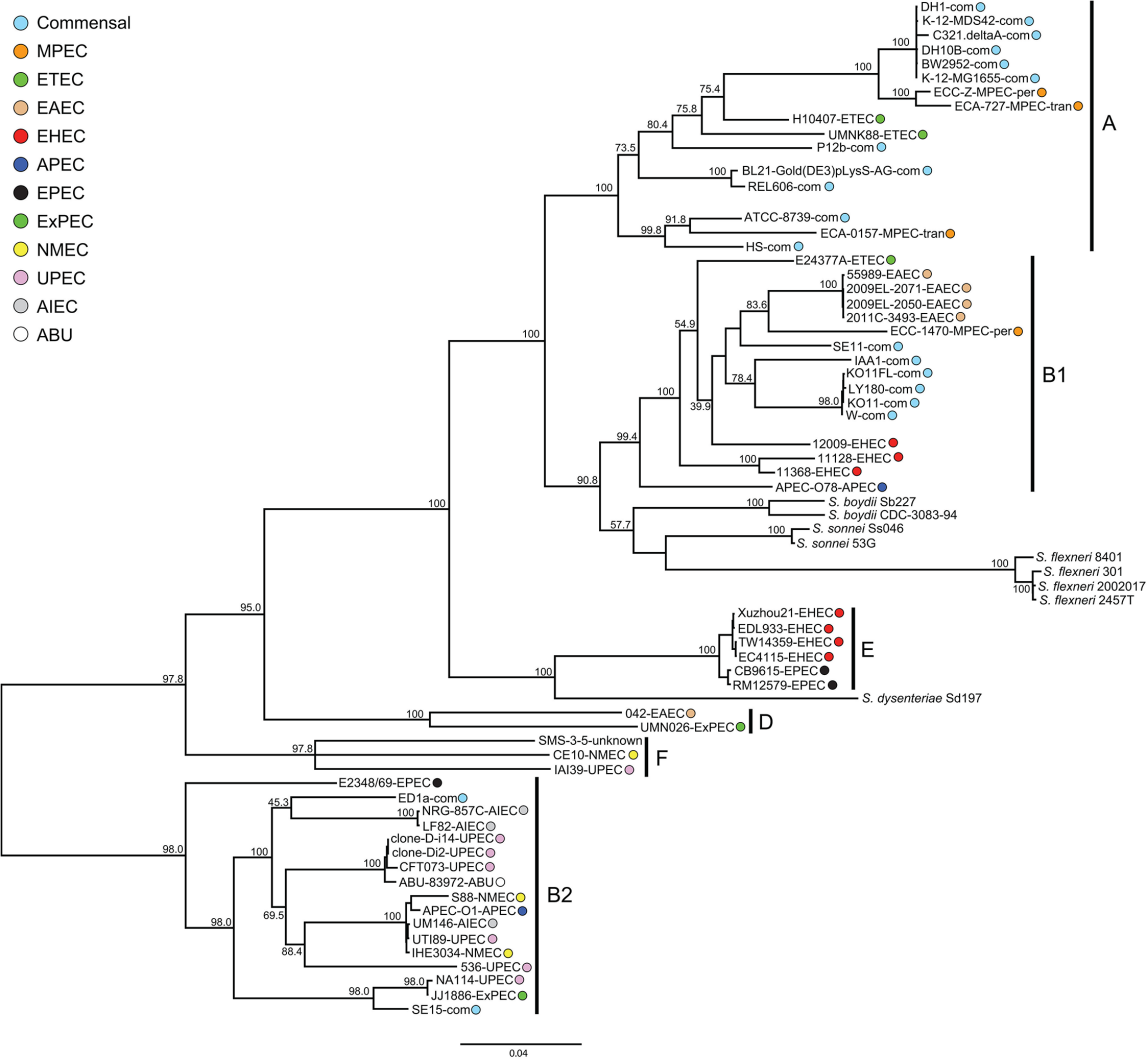
Maximum likelihood phylogeny generated from 16,062 SNPs. Bootstrap support values are shown over branches. Branch lengths are drawn proportion to the amount of sequence change. Phylogroup membership is indicated with a horizontal bar and the corresponding letter. Pathotypes of each of the *E*. *coli* strains is indicated with a color code and their lettered abbreviation adjacent to the strain names of each; commensal strains are abbreviated as “com”.

**Fig 2:**
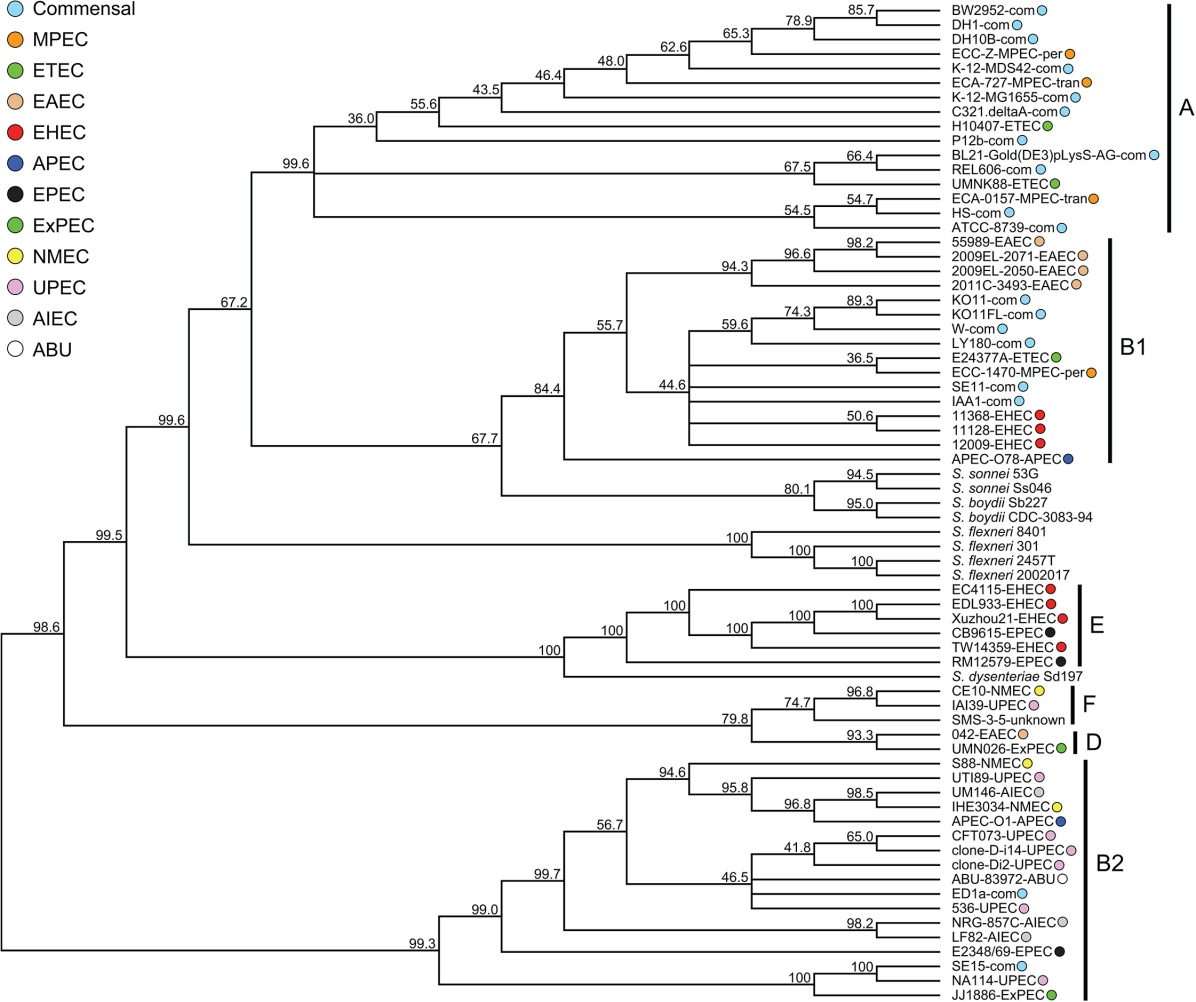
Consensus of 438 maximum likelihood gene phylogenies. Proportion of genes supporting groups shown on branches. Phylogroup and pathotype information same as in Fig 1.

**Fig 3:**
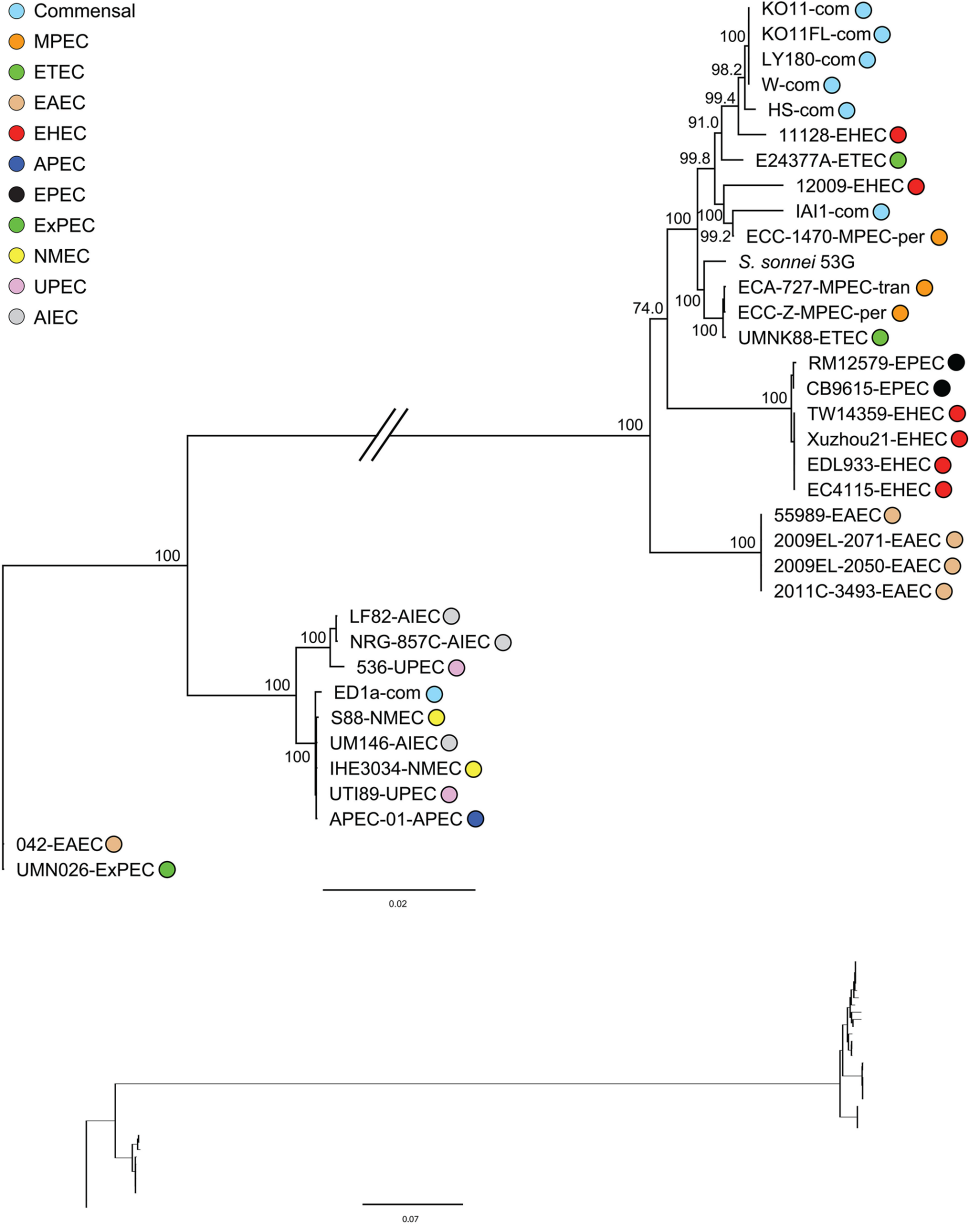
Maximum likelihood phylogeny for type six secretion system. Bootstrap support values are shown over branches. Branch lengths drawn proportion to the amount of sequence change. Phylogroup and pathotype information same as in Fig 1.
